# Impact of a mobile reference application on the efficiency and accuracy of cardiac implantable electronic device data retrieval: a comparative pilot study

**DOI:** 10.1093/europace/euag206

**Published:** 2026-08-11

**Authors:** Matteo Ziacchi, Sergio Datteri, Serge Boveda, Sakis Themistoclakis, Pascal Defaye, Giuseppe Coppola, Giulio Molon, Domenico Della Rocca, Matteo Bertini, Jean Claude Deharo, Federico Migliore, Giuseppe Sgarito, Martina Nesti, Christophe Leclercq, Mauro Biffi

**Affiliations:** Institute of Cardiology, IRCCS Azienda Ospedaliero Universitaria di Bologna, Bologna, Italy; iPacemaker, Milan, Italy; Division of Cardiology, Clinic Pasteur, Toulouse, France; Division of Cardiology, Hospital dell’Angelo, Mestre-Venice, Italy; Division of Cardiology, Grenoble Alpes University Hospital, Grenoble, France; I.C.U. Cardiology-Ep Lab, University Hospital ″Paolo Giaccone″, Palermo, Italy; Division of Cardiology, IRCSS Sacro Cuore-Don Calabria Hospital, Negrar, Italy; Division of Cardiology, UZ Brussel, Bruxelles, Belgium; Division of Cardiology, University Hospital of Ferrara, Ferrara, Italy; Division of Cardiology, APHM La Timone Hospital, Marseille, France; Department of Cardiac, Thoracic Vascular Sciences and Public Health, University of Padova, Italy; Division of Cardiology, IRCCS ISMETT-UPMC Palermo, Palermo, Italy; Department of Electrophysiology, Fondazione Toscana Gabriele Monasterio, Pisa, Italy; Division of Cardiology Hospital Pontchaillou of Rennes, Rennes, France; Institute of Cardiology, IRCCS Azienda Ospedaliero Universitaria di Bologna, Bologna, Italy

## Introduction and purpose

Rapid access to reliable technical information on cardiac implantable electronic devices (CIEDs) is essential during implantation, follow-up, and troubleshooting.^1^ However, conventional sources such as manuals, technical PDFs, and manufacturer websites are often fragmented, time-consuming, and prone to interpretation errors.

Device therapy has evolved considerably in recent years. The progressive diffusion of conduction system pacing, MRI-conditional systems, leadless and extravascular technologies, and remote monitoring has increased both the volume and the heterogeneity of the technical information that has to be retrieved at the point of care.^2–4^ Each device–lead combination carries its own technical profile, dispersed across manufacturer-specific sources.

Digital health and artificial intelligence (AI) have introduced new approaches to information retrieval and digital clinical support. A recent European Heart Rhythm Association perspective has highlighted the accelerating implementation of digital tools and AI in electrophysiology while emphasizing that data quality, transparency, and clinical validation remain prerequisites for their safe adoption.^5,6^ The reliability of general-purpose AI systems in this highly specialized domain nevertheless remains uncertain, particularly when complex structured datasets are involved.

This study aimed to compare conventional methods, AI-assisted search (AIs), and a dedicated mobile application (MA) for retrieval of technical CIED information.

## Methods

This multicentre pilot study involved physicians and allied professionals experienced in CIED management.

Participants were asked to answer four categories of structured technical questions:

- Device connector identification
- Theoretical device longevity
- MRI compatibility of device–lead systems
- Retrieval of official advisories

Questions were organized into four randomly generated datasets, each containing one question per category, to minimize learning bias.

Each participant completed the assessment using three different modalities in a predefined sequence:

- Conventional methods (manuals, PDFs, and web search)
- Dedicated mobile application—iPacemaker
- AI-assisted search using ChatGPT, an AI assistant developed by OpenAI

**Figure 1:**
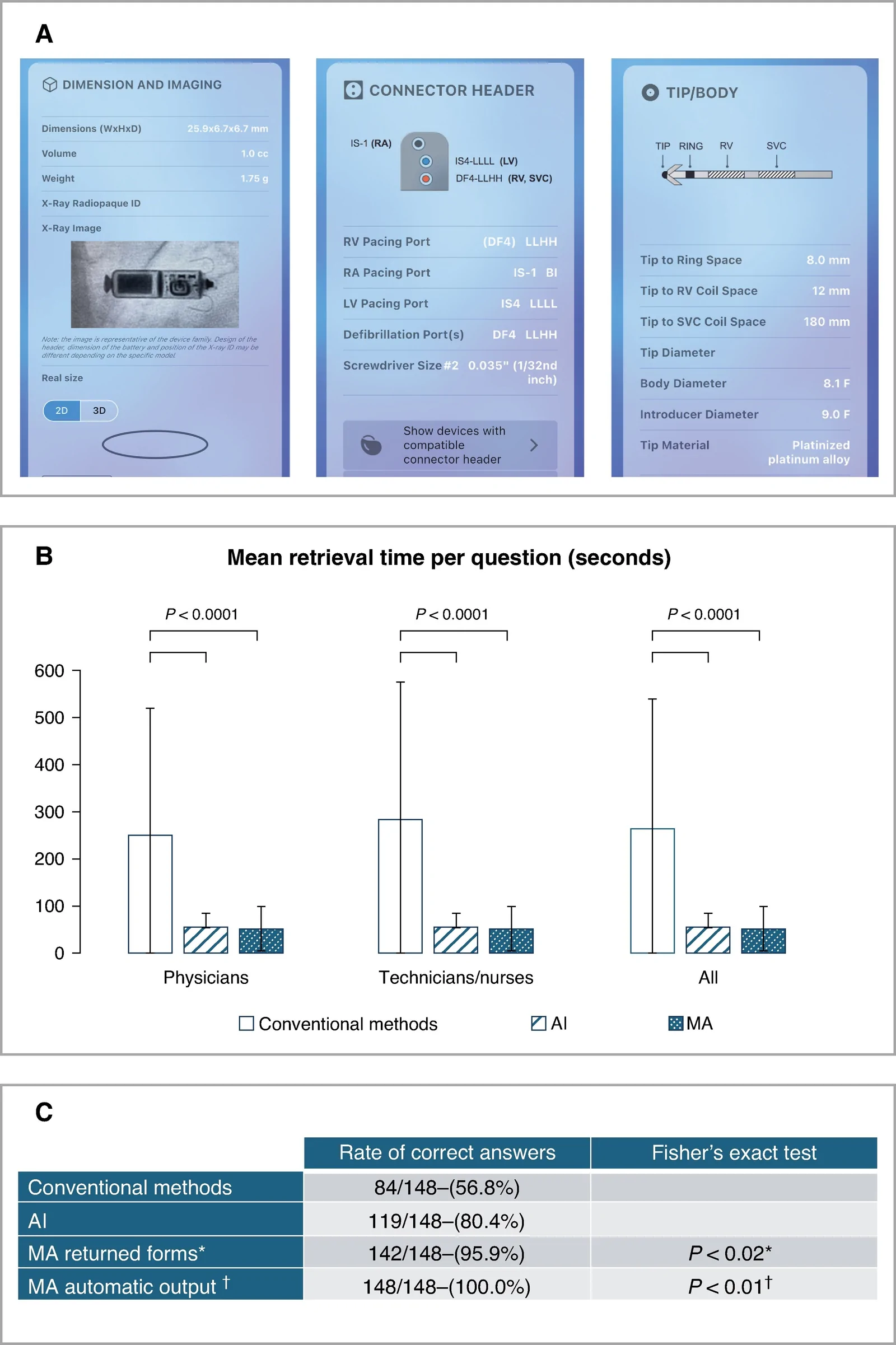
Comparison of conventional methods, AI-assisted search, and a structured mobile reference application for retrieval of technical CIED information. (*A*) Representative screenshots of the mobile application showing structured technical information including device dimensions, radiographic identification, connector configuration, and lead characteristics. (*B*) Mean retrieval time per question using conventional methods, AI-assisted search, and MA. Error bars indicate standard deviation. Overall differences were analysed using one-way ANOVA; *P* values for the comparison vs. conventional methods are reported in the panel; MA vs. AI-assisted search, *P* = 0.98. (*C*) Rate of correct answers obtained using conventional methods, AI-assisted search, and the mobile application (returned forms and automatic output). Asterisk (*) indicates the comparison between MA returned forms and AI-assisted search; Dagger (†) indicates the comparison between MA automatic output and AI-assisted search. All comparisons of accuracy were performed using Fisher’s exact test. Abbreviations: AI, artificial intelligence; ANOVA, analysis of variance; CIED, cardiac implantable electronic device; MA, mobile application; MRI, magnetic resonance imaging; SD, standard deviation.

iPacemaker is a commercially available mobile reference application containing a structured database of technical CIED information, including connector configurations, theoretical longevity, MRI compatibility, lead characteristics, radiographic identification, and manufacturer advisories, covering more than 7000 devices from over 60 manufacturers. Data are curated from official manufacturer documentation and organized in a standardized, queryable format (*Figure 1A*).

Each modality was applied to a different randomly assigned dataset, ensuring that no participant encountered the same question twice.

For each question, retrieval time was recorded up to 15 min, and accuracy was assessed against official manufacturer documentation. The time limit was intended to reflect clinical practice. Time to complete the tasks was analysed using one-way analysis of variance (ANOVA), while answer accuracy was compared using Fisher’s exact test.

For the mobile application, accuracy was evaluated both from the answers entered by participants on the returned forms and from the corresponding information displayed automatically by the application. This distinction allowed user transcription or interpretation errors to be separated from database output errors.

## Results

The study included 18 hospitals across Italy and France, involving 24 physicians and 13 technicians/nurses experienced in CIED management.

Mean retrieval time per question was significantly shorter using both the MA and AIs than conventional methods (*P* < 0.0001), whereas no significant difference was observed between MA and AIs (*P* = 0.98) (*Figure 1B*). Mean retrieval times were 264 ± 277 s with conventional methods, 56 ± 29 s with AI-assisted search, and 53 ± 44 s with the mobile application. No participant exceeded the predefined 15-min limit when using either digital tool.

The mobile application achieved the highest accuracy (*Figure 1C*). Participant-completed answer forms contained 142/148 (95.9%) correct responses, whereas the application’s automatic output correctly answered all 148 questions (100%). AI-assisted search correctly answered 119/148 questions (80.4%), and conventional methods 84/148 (56.8%). Qualitative review showed that incorrect AI-generated answers were generally expressed in a confident and affirmative form and were concentrated in questions requiring the integration of multiple technical variables, such as device–lead MRI compatibility, connector configurations, and theoretical longevity.

## Discussion

Both AI-assisted search and the structured mobile reference application substantially reduced information retrieval time compared with conventional methods. However, rapid access alone is insufficient in clinical practice, where accuracy, traceability, and reproducibility are equally important.

The pattern of AI errors observed here appears clinically relevant. Incorrect responses were not flagged by any uncertainty marker and were phrased as plausibly as correct ones, so detection required independent verification against the source documentation, thereby reducing the practical benefit of rapid information retrieval. These observations are consistent with previously reported limitations of large language models in specialized medical applications, including the generation of coherent but unfounded content.^7,8^ The 80.4% accuracy observed with AI-assisted search should also be interpreted cautiously, since it was partly sustained by question types, such as advisory retrieval, for which information is readily available online.

Conversely, the structured mobile application provided consistently accurate information. Its database was derived from curated manufacturer documentation and presented in a standardized format rather than generated probabilistically at the time of the query. The few incorrect responses recorded in the returned forms reflected user transcription or interpretation errors by first-time, previously unexposed users rather than database inaccuracies. It is also likely that the 15-min cap underestimates the real-world inefficiency of conventional methods, since complex CIED-related queries may require considerably longer retrieval times in daily practice.

These findings should be interpreted within the broader digital transformation in electrophysiology. Digital decision-support systems and AI are expected to play an increasingly important role in arrhythmia management, provided that reliability, transparency, and clinical validation remain central to their implementation.^6^ The present data suggest that, for the retrieval of technical CIED information, structured domain-specific databases currently offer greater reliability than general-purpose AI systems and that the two approaches address different needs rather than competing directly. Limitations include the pilot design, the limited number of questions per participant, and the single AI system tested.

## Conclusions

A dedicated mobile reference application allowed faster and more accurate retrieval of technical CIED information than conventional methods and showed higher accuracy than AI-assisted search. These findings support the use of structured digital reference tools in clinical electrophysiology practice, where rapid access to reliable technical information is important.

## Data Availability

The data underlying this article are available from the corresponding author upon reasonable request.
